# Visuospatial Attention to Single and Multiple Objects Is Independently Impaired in Parkinson's Disease

**DOI:** 10.1371/journal.pone.0150013

**Published:** 2016-03-10

**Authors:** Daniel J. Norton, Victoria A. Nguyen, Michaela F. Lewis, Gretchen O. Reynolds, David C. Somers, Alice Cronin-Golomb

**Affiliations:** 1 Department of Psychiatry, Massachusetts General Hospital and Harvard Medical School, Boston, Massachusetts, United States of America; 2 Department of Psychological and Brain Sciences, Boston University, Boston, Massachusetts, United States of America; 3 Department of Neuroscience, Brown University, Providence, Rhode Island, United States of America; Centre de Neuroscience Cognitive, FRANCE

## Abstract

Parkinson’s disease (PD) is associated with deficits in visuospatial attention. It is as yet unknown whether these attentional deficits begin at a perceptual level or instead reflect disruptions in oculomotor or higher-order processes. In the present study, non-demented individuals with PD and matched normal control adults (NC) participated in two tasks requiring sustained visuospatial attention, both based on a multiple object tracking paradigm. Eye tracking was used to ensure central fixation. In Experiment 1 (26 PD, 21 NC), a pair of identical red dots (one target, one distractor) rotated randomly for three seconds at varied speeds. The task was to maintain the identity of the sole target, which was labeled prior to each trial. PD were less accurate than NC overall (p = .049). When considering only trials where fixation was maintained, however, there was no significant group difference, suggesting that the deficit’s origin is closely related to oculomotor processing. To determine whether PD had additional impairment in multifocal attention, in Experiment 2 (25 PD, 15 NC), two targets were presented along with distractors at a moderate speed, along with a control condition in which dots remained stationary. PD were less accurate than NC for moving (p = 0.02) but not stationary targets. This group difference remained significant when considering only trials where fixation was maintained, suggesting the source of the PD deficit was independent from oculomotor processing. Taken together, the results implicate separate mechanisms for single vs. multiple object tracking deficits in PD.

## Introduction

While Parkinson’s disease (PD) is primarily considered a motor disorder, cognitive and perceptual disturbances also occur and have a substantial impact on the quality of life beyond the disease’s classic motor symptoms [1–3] One such perceptual-cognitive function that is compromised in PD is visuospatial attention. Researchers have found that visuospatial attention, elicited by a cue of some kind, is intact in PD, but that individuals with PD disengage their attention more quickly than healthy adults, and have weakened inhibition of return (IoR); [4–6]. Sustained visual attention, however, has not been studied in PD, and is also important for real-world activities such as driving. In addition, limitations of the previous studies on visuospatial attention in PD leave important questions unanswered.

A principal limitation of the current literature on attention in PD is that most studies have required motor execution in performing the task (e.g., pressing a button as quickly as possible in response to a stimulus). This raises the question as to whether individuals with PD are impaired in their visuospatial attention at a low (perceptual) level, or instead whether their impaired motor system has difficulty taking advantage of a normally functioning attention system. A second limitation is that these studies all measured attention rather indirectly, using subtle changes in reaction time or perceptual performance in cue- versus no-cue conditions. These effects are often small and intrinsically linked to noise from the non-attention variables to which they are tied, making it difficult to isolate and understand changes only in perceptual ability. For example, one recent study attempted to assess covert visuospatial attention in PD by measuring the effect of attention drawn by a cue upon contrast sensitivity, but attention did not affect contrast sensitivity in either the control or the PD group [7]. Therefore, using improvements in contrast sensitivity or reaction time to indirectly measure attention may not accurately identify changes in visuospatial attention that are in fact due to PD.

Multiple object tracking (MOT), which measures sustained attention, is an attractive paradigm for studying visuospatial attention in PD, because motor behavior (such as quickly pressing a key) is not critical to the results, and because attentional capacity determines the observer’s accuracy on the task. Here, spatial attention is spread over multiple targets that move among identical distractors [8]. The task is to keep track of the target dots (which are indicated at the beginning of each trial) as they move around distractor dots. Unless one successfully deploys attention to the targets, one will lose track of them among the distractors. Observers are limited in their ability to track dots based on the speed, number and spatial characteristics (e.g., proximity to distractors) [9,10]. This task has been shown to be sensitive to healthy aging and clinical disorders, adding to the promise of applying it to PD [8,11 12]. The present study adapted this paradigm to characterize visuospatial attention to a single object or to multiple objects simultaneously.

Use of the MOT paradigm in PD can also provide information on the integrity of the brain’s attentional systems in this disorder. PD is a heterogeneous disease that affects several brain regions that play key roles in attention, including parietal cortex, prefrontal cortex, and the basal ganglia [13–15]. Prior work indicates that while tracking even a single object activates a wide network of brain regions, only a subset of these regions is sensitive to the number of objects tracked [16,17]. If PD patients show a deficit in tracking a single object, and this is tightly correlated with tracking multiple objects, it would support the idea that these stem from the same mechanism. If, on the other hand, tracking one object is not correlated with tracking two or more objects in PD, then it can be inferred that distinct processes are impacted.

In Experiment 1, we measured performance in PD and age-matched control adults while tracking a single object as a function of the speed of rotation of a target dot with a distractor dot with which it was paired. Attended objects can only be shuffled about among distractors at a certain maximum frequency before one loses track of the target [18]. On the basis of shortened sustained covert attention in PD [6], we hypothesized that PD would be less accurate than NC in tracking a single object.

In Experiment 2, we measured multifocal attention in PD and NC as a function of spatial arrangement of the stimuli. Individuals are able to deploy spatial attention to multiple locations at once [8], but are limited in the number of objects they can accurately track. Of note, the resources for tracking exhibit a strong degree of independence between left and right visual hemifields, meaning that nearly twice the number of objects can be tracked at once if they are distributed across hemifields as opposed to within a hemifield [19]. In Experiment 2, we took advantage of the latter characteristic of spatial attention to test a hypothesis about attentional processing in PD. Rather than measuring accuracy while tracking four objects at once (which can be intimidating for some observers), we used only two objects at once, but presented them in the same vs. opposite hemifields. We anticipated that PD would show similar impairment in the condition where the targets were in opposite hemifields as they showed in Experiment 1, and that these results would be correlated, because they should amount to the same task (with each hemifield independently tracking one object). We anticipated that there we also be a deficit in tracking multiple objects within a hemifield because of diminished sustained attention in general. We examined the performance data from both tasks in its raw form, and also filtering out trials where subjects did not maintain fixation (determined using eye-tracking).

Another aspect of our analysis probed spatial neglect in PD, which, if present, might be expected to bring about differential impairment in the left hemifield in the affected individuals. Some studies [20–22] have suggested that spatial neglect may exist particularly in those for whom the disease began on the left side of the body (LPD). This is consistnet with the well-established position that the more affected hemisphere in LPD (the right), is especially important for spatial attention [23]. We selected our groups of LPD/RPD based on initial onset because PD remains neurobiologically asymmetrical throughout individuals’ lives, even when motor symptoms become bilateral [24,25] with the originally affected side remaining the more impaired side, even when examining the brain for pathology at autopsy. In addition, we also examined results with respect to asymmetry of current motor symptoms. Up to this point, attention has not been directly measured in the left versus right hemifield in LPD except through visual exploration tasks [26]. The present study presented MOT stimuli in the left and right periphery to ensure presentation of the stimuli to the left and right hemifields. We anticipated that LPD would show differential impairment in tracking objects in the left hemifield, particularly in Experiment 2 when there was a distractor in the right hemifield, or when the load in the left hemifield was high (tracking two objects at once).

## Experiment 1 Methods

### Participants

Twenty-six non-demented individuals with idiopathic PD (12 LPD and 14 RPD) and 21 normal control adults (NC) participated in the study. Demographic and other participant information is shown in Table 1; the groups were matched on age, education, male:female ratio, and premorbid intelligence as measured by the vocabulary section of the Wide Range Achievement Test [27]. Potential participants were excluded from the study on the basis of having neurological conditions (other than PD, for the PD group), coexisting serious chronic medical illnesses including psychiatric illness, use of psychoactive medication besides antidepressants and anxiolytics in the PD group, history of intracranial surgery (e.g., deep brain stimulation or other invasive PD treatments), traumatic brain injury, and current alcohol dependence or substance abuse. When possible, participants received a detailed neuro-ophthalmological examination to rule out visual disorders including significant glaucoma, cataracts, and macular degeneration. They were screened for dementia using the Columbia Modified Mini-Mental State Examination (MMSE) [28]; scores were converted to standard MMSE scale, and the minimum MMSE score for inclusion in the study was a 27 out of a possible total score of 30. Disease stage and severity was mild to moderate as indicated by the range of scores on the Hoehn and Yahr scale (1 to 3; median 2) and the mean UPDRS motor score of 17.5 [29,30]. The Beck Depression Inventory II and Beck Anxiety Inventory were administered to evaluate potential effects of mood on the dependent variables [31,32]. This research project was approved by the Boston University Institutional Review Board (IRB), and was conducted according to the principles expressed in the Declaration of Helsinki. Written informed consent was obtained from all participants.

**Table 1:** Participant Characteristics.

Measure	PD (n = 26)	LPD (n = 12)	RPD (n = 14)	NC (*n* = 21)	Significance PD vs. NC
Age (years)	63.5 (6.4)	63.5 (6.8)	63.6 (6.3)	67.4 (7.6)	*NS*
Education (years)	17.1 (1.8)	17.1 (2.2)	17.1 (1.3)	17.3 (1.8)	*NS*
Male/Female	11/15	6/6	5/9	6/15	*NS*
UPDRS Motor Score	17.57 (7.3)	17.8 (6.7)	17.4 (7.9)	—	*—*
H & Y Stage	1.9 (0.6)	1.6 (0.5)	2.1 (0.5)	—	*—*
LED (mg/day)	465.6 (286)	396.8 (237.8)	524.6 (304.8)	—	*—*
Acuity (decimal)	.85 (.20)	.75 (.20)	.92 (.18)	.87 (.23)	*NS*
BDI-II	6 (4.9)	5.0 (4.0)	6.9 (5.7)	3.3 (3.7)	*p<0*.*05*
BAI	6.4 (4.8)	4.5 (3.3)	8 (5.3)	3.2 (4.3)	*p<0*.*05*

*Note*. LPD = left-onset Parkinson’s disease; RPD = right-onset Parkinson’s disease; NC = normal control participants. UPDRS = Unified Parkinson’s Disease Rating Scale; H & Y = Hoehn & Yahr staging criteria; LED = Levodopa equivalent dosage (Tomlinson et al., 2010); BDI-II = Beck Depression Inventory– 2; BAI = Beck Anxiety Inventory. Values presented are means (standard deviations), unless otherwise indicated.

### Stimuli and procedures

**Fig 1:**
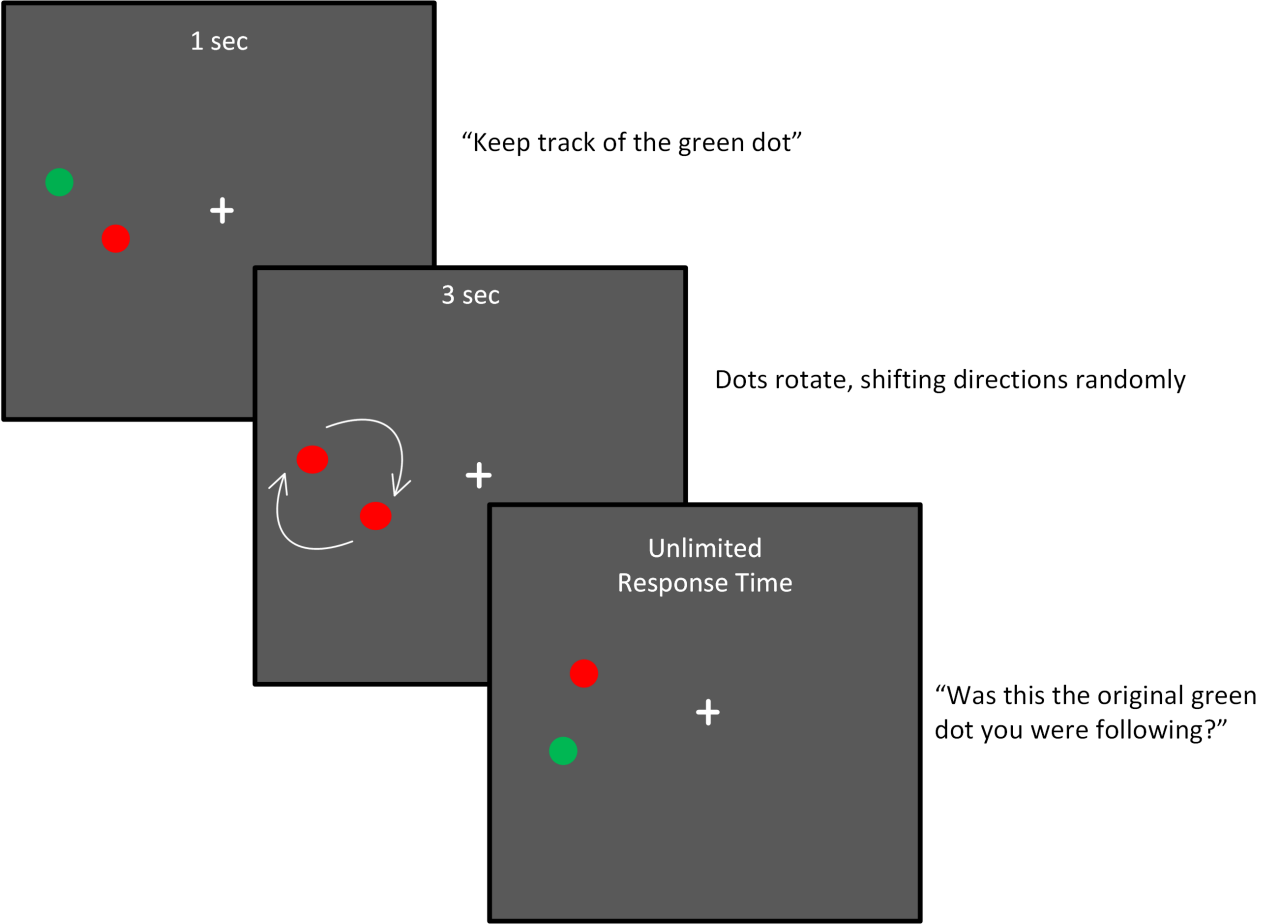
Methods for Experiment 1. Dots rotated at one of four speeds as described in the text, and were placed on either the left or the right of the fixation cross on each trial.

Participants were presented a pair of red dots rotating around a central location, as shown in Fig 1. To identify the target, one of the dots briefly appeared green before motion onset, and then it turned to red like its partner so that the two could no longer be distinguished on the basis of any visual feature besides location. Participants maintained fixation on a central cross during the task, and the dot pair was presented with its center 9.2 degrees to the left or right of the fixation target. The dots rotated either counter clockwise or clockwise for a quarter of a full rotation. The direction of the dots was reset every quarter turn randomly, so that they could either proceed in the same direction as the previous quarter turn, or could reverse direction. In this way, the only way to maintain knowledge of which dot was the target was to follow it with one’s “attentional spotlight”. After three seconds, the dot motion stopped, and one of the dots turned green. The task was to indicate whether this green dot at the end of the trial was the same dot as the green target in the beginning of the trial. Dots subtended 0.54 degrees of visual angle, and rotated with a radius of 1.8 degrees from the center point. The speed of the dots was set to be 4.3, 8.5, 17.2 or 34 degrees/sec. There were eight trials at each speed on each side of the screen. Of those eight trials, four had the target dot probed at the end, and four had the distractor dot probed at the end. The total was 64 trials (4 repetitions X four speeds X two probe conditions X two positions). The main outcome measure was accuracy at each speed. In order to compare performance across experiments, we also calculated a summary variable for Experiment 1 in the form of a speed threshold. This was the speed of rotation at which subjects could perform the task at 80% accuracy. This was done by fitting the four accuracies to a Weibul function of the form $y=1-0.5\;*\;e^{{\left(\frac{-x}{a}\right)}^{b}}$ where *y* is the proportion correct, *x* is the speed of rotation, and *a* and *b* are curve fitting parameters. Stimuli were programmed using Psychophysics Toolbox and MATLAB [33] and were presented on a 21” CRT monitor (Hewlett Packard FP2141sb.) running at 120 Hz.

### Eye tracking

An Applied Science Laboratories Eye-Trac 6 camera was used to investigate maintenance of fixation during the task. The manufacturer reports that the system accuracy is 0.5 deg of visual angle, and the resolution is .25 deg of visual angle. Eye gaze was recorded during each trial at 120 Hz. Performance in tracking single or multiple objects is known to be enhanced by moving one’s gaze to follow a single object or the centroid of multiple objects [34]. On the other hand, for rapidly moving targets, planning and executing a saccade could cause one to lose track of the target and its distractor. Therefore, it is critical to carefully evaluate the extent to which any group differences in task performance should be attributed to differing patterns of eye movements between the groups. Toward this end, we re-analyzed the performance data for the main analyses, filtering out trials where subjects did not maintain fixation. For these secondary analyses, trials were thrown out if 1) they contained a saccade (defined as velocity, averaged across 5 frames, exceeding 100 deg/sec at any point during stimulus motion), 2) the subject looked outside a square window of 8.4 degrees of visual angle in height and width, centered on the fixation cross, or where greater than 50% of the eye data were lost (due to poor lock on the eye). For some subjects, this filtering resulted in a significant reduction of the sample size for particular conditions. We threw out data points that were representative of fewer than 4 trials. For example, if one subject had only 2 left-trials and 1 right-trial for speed 1, after removing trials where fixation was broken, we would throw out that point as unreliable. We then replaced the missing data using imputed datasets, where we predicted the missing values using a regression model that included unfiltered accuracy, group, and proportion of trials lost due to broken fixation. In addition to this re-analysis of the accuracy data, we also compared PD and NC directly on the proportion of trials on which fixation was successfully maintained.

## Experiment 1: Results

### PD vs. NC

**Fig 2:**
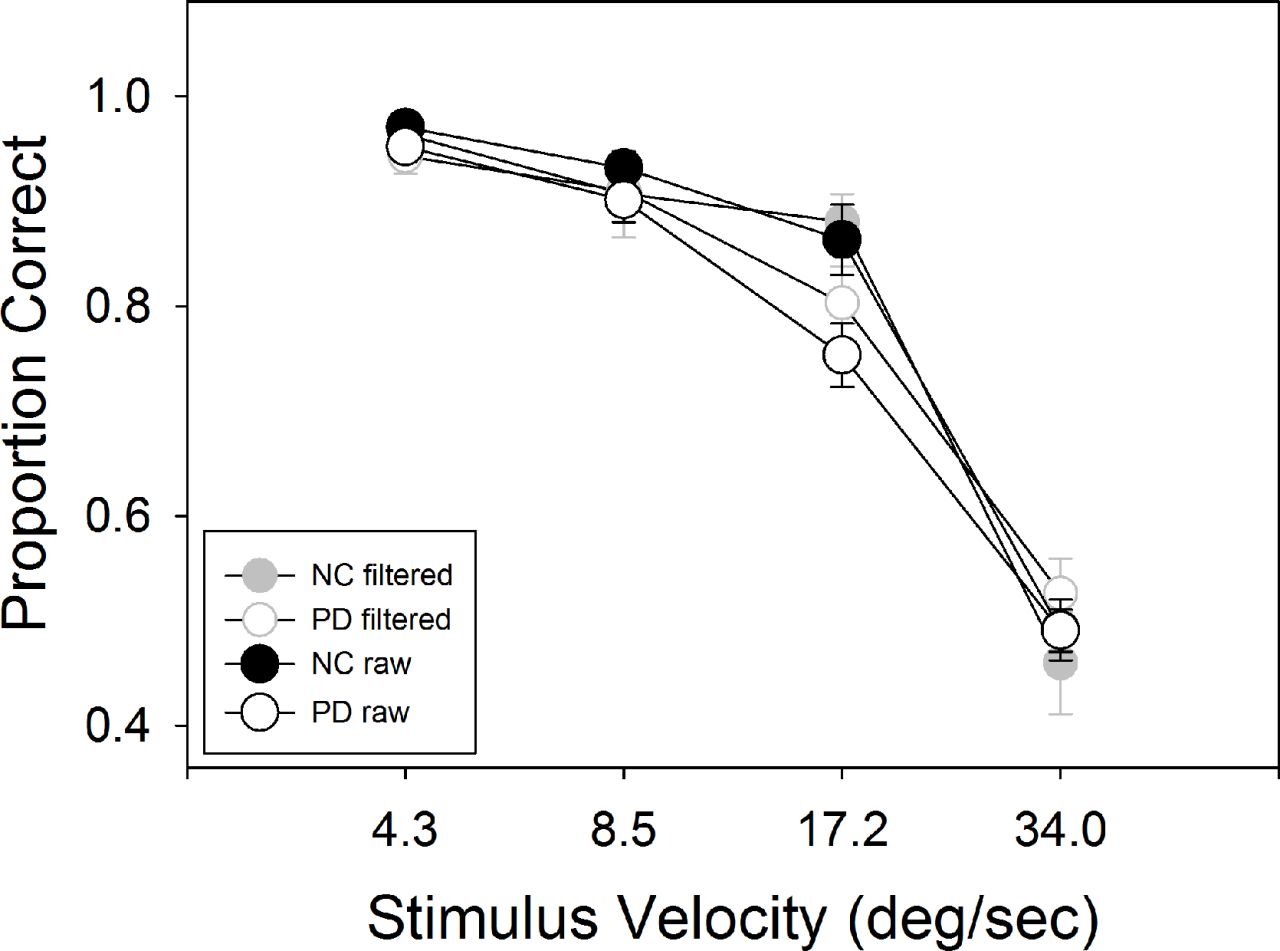
Results of Experiment 1. PD showed lower accuracy than NC overall (p = .049) for the raw dataset, but not when using the filtered dataset based on only trials where subjects maintained fixation.

Results for Experiment 1 are shown in Fig 2. A two-way ANOVA, across four speeds comparing PD vs. NC, showed main effects for group, F (1, 45) = 4.09, p = .049, η^2^ = .083, and speed, F(2.22, 100.01) = 219.66, p < .001, η^2^ = .83. There was no interaction between the two, F (2.29, 100.01) = 2.29, p = .101, η^2^ = .048. A t-test comparing speed thresholds between groups was also significant, t(45) = 2.57, p = .013. We repeated this analysis using only the trials where subjects maintained fixation. 6.9% of the data were missing due to their being representative of fewer than 4 trials, and were replaced using multiple imputations as described in the methods. We ran 25 separate ANOVA’s, one for each imputed dataset. The average main effect of speed was significant at mean F = 104.1, (95% CI: 100.74–107.46), mean p < .001, mean η^2^ = .697 (95% CI: .69–.704), but the average main effect for group was not, mean F = .13 (95% CI: = .075–.194), mean p = .76 (95% CI: .70–.81), mean η^2^ = .003 (95% CI: .002–.004), and there was no interaction between the two, mean F = 2.09 (95% CI: 1.77–2.40), mean p = .15, (95%CI: .11–.19), mean η^2^ = .06 (95% CI: .02–.11). We also compared the speed thresholds based on the filtered accuracy data (where we took the mean of the 25 imputed accuracy datasets for each subject and speed to compute the thresholds based on trials where fixation was maintained). Similarly to the filtered accuracy data, thresholds were also no longer different between PD and NC, t(45) = .74, p = .47.

**Fig 3:**
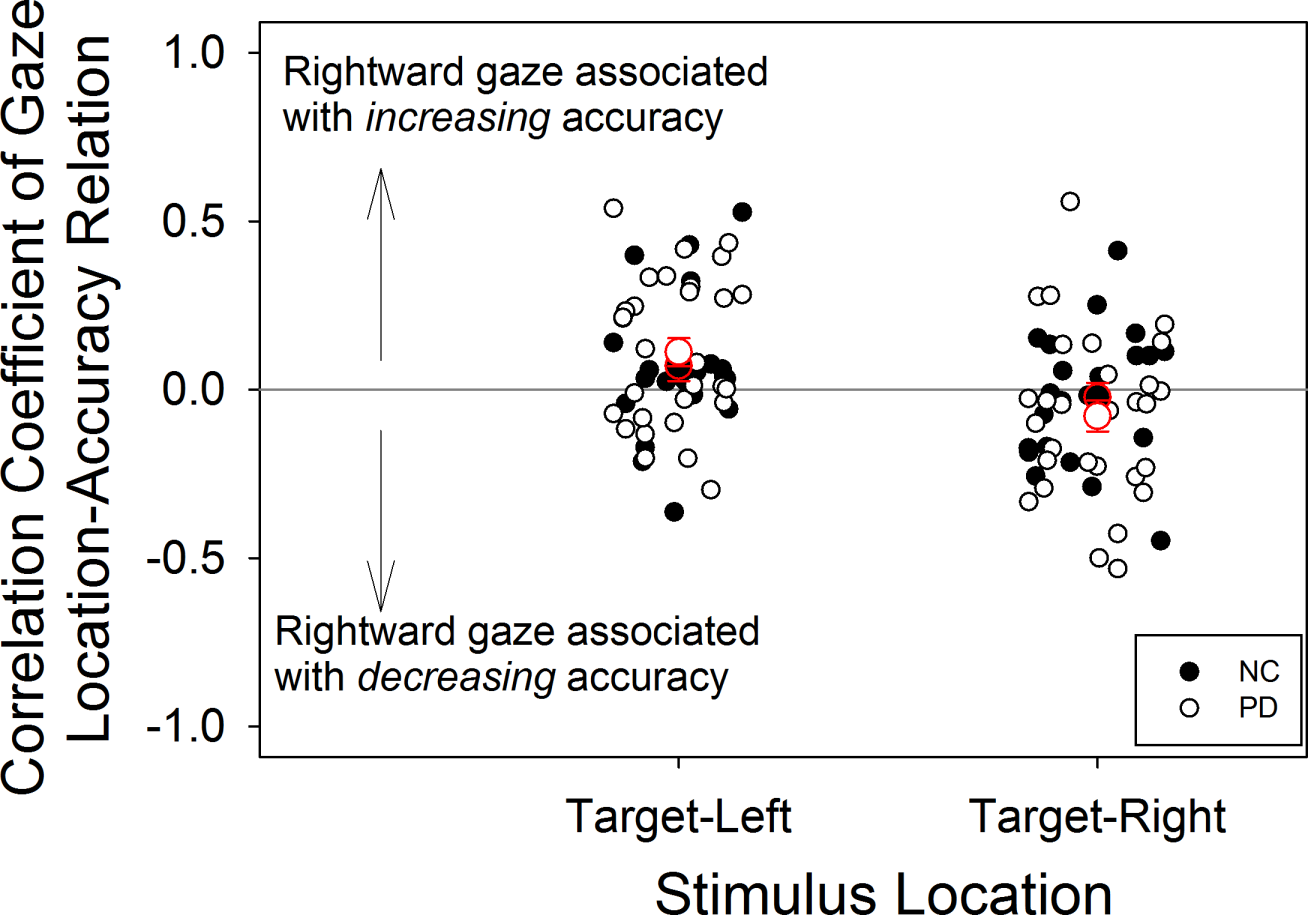
Relation between gaze location and accuracy for experiment 1. Group averages are shown with red outlines, and these indicate that the relation between gaze location and accuracy across subjects was weak, despite some medium strength correlations in individual subjects.

Comparing groups on the proportion of trials in which fixation was successfully maintained, a mixed ANOVA on group and speed showed no group difference, F(1,45) = .34, p = .56, η^2^ = .01, but the effect of speed was significant, F(3,135) = 6.3, p = .001, η^2^ = .12, as was the interaction between group and speed F(3,135) = 5.2, p = .002, η^2^ = .10. We also examined the relation between accuracy and gaze location for individual subjects (Fig 3). For each subject, accuracy on each trial (coded as 0 or 1) was regressed as the dependent variable upon the average horizontal coordinate of the gaze location for that trial as the independent variable. Positive values indicated that, for an individual subject, rightward gaze was associated with increased accuracy, and negative values the opposite. Trials with the stimulus on the left and those with the stimulus on the right were analyzed separately. PD showed, on average, no correlation between accuracy and proximity of gaze to the stimulus on the horizontal axis (averaged r = .09, SD = .23). NC showed the same lack of correlation (average r = .05, SD = .19).

BAI and BDI were not correlated with averaged accuracy on this task in the PD or the NC group (p > 0.05).

### LPD analysis

A three-way ANOVA across side of screen, group (LPD n = 12; RPD n = 14; or NC n = 21) and speed showed a main effect for speed, F(2.2, 100) = 204, p < .001, η^2^ = .82, but not for group, F(2,44) = 2.0, p = .15, η^2^ = .08, or side of screen F(1,44) = .04, p = .91, η^2^ < .01. There were no significant interactions between any of these three variables.

## Experiment 2: Methods

### Participants

Participants were a subset of those participating in Experiment 1, and included 25 PD (12 LPD and 13 RPD) and 15 NC.

### Stimuli and Procedures

**Fig 4:**
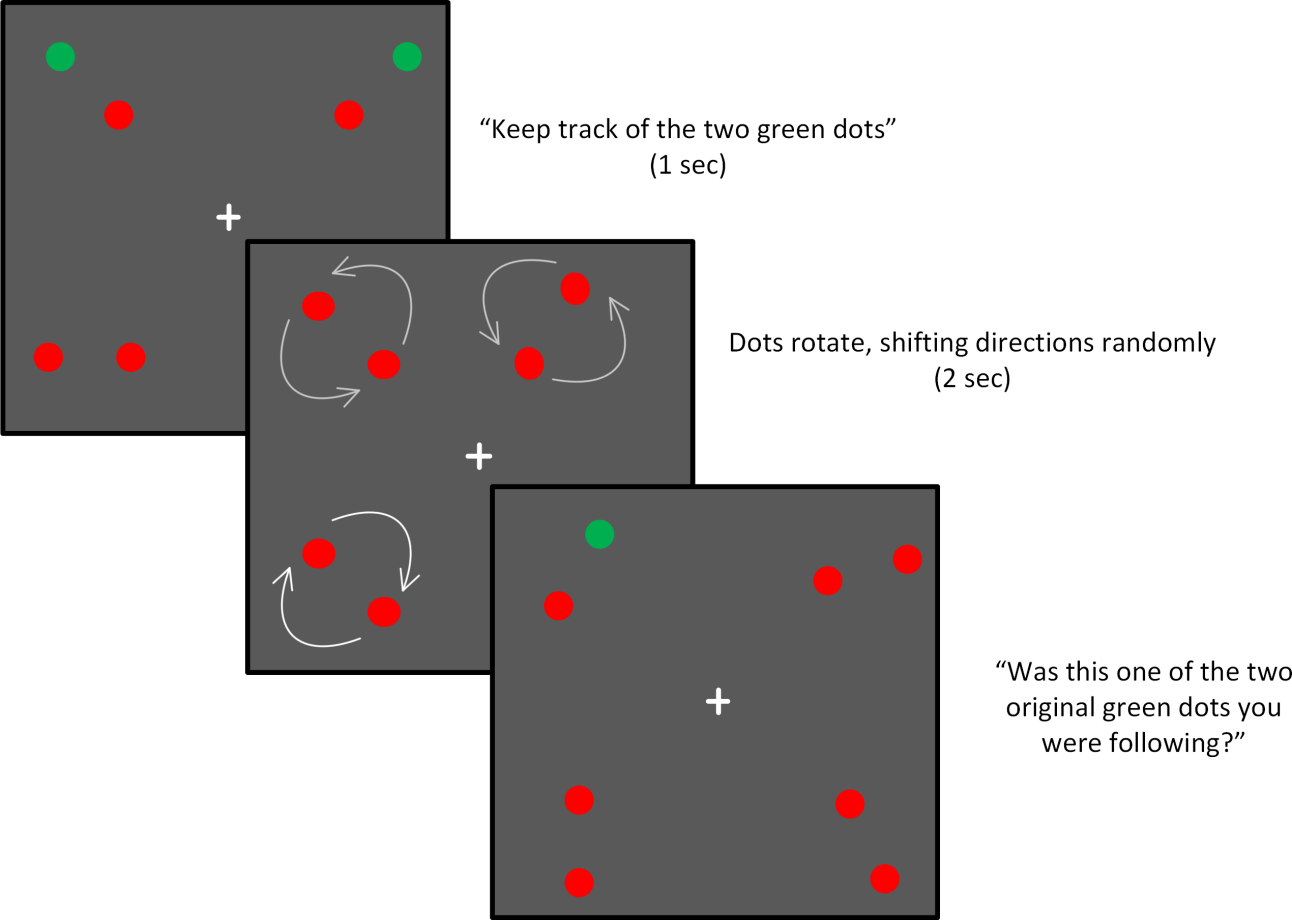
Stimuli and task for Experiment 2. In this experiment, dots rotated at a constant, moderate speed. The critical variable was the spatial arrangement of the two targets, which were placed in either the same hemifield (SH) or opposite hemifield (OH) as each other.

Procedures were the same as in Experiment 1 except as follows: First, speed was set to 12.8 deg/sec for all trials. This value was selected based on pilot data indicating that this was a speed at which the task was challenging but doable for most participants. Second, there were four dot-pairs presented each trial, one in each quadrant of the screen (Fig 4). Two of the dot pairs contained a target dot, and participants were instructed to allocate their attention to both of them. Only one dot was probed at the end of the trial: either one of the two target dots, or a distractor dot from the same two pairs. Third, the dots rotated for only 2.1 seconds, rather than 3.0 duration used in the first experiment. A task demonstration was done to ensure that the participants understood the instructions.

Since this task is often experienced as very difficult and somewhat overwhelming for many participants (regardless of group), an additional condition was added to ensure that they were capable of remembering which dots were the targets, apart from their movement. In this condition, the task was the same as in the main condition, but the dots did not rotate at all. In addition to providing a control for working memory components of the task, this condition also served as reinforcement for participants who were struggling during the test—occasionally they could provide the correct answer confidently.

With respect to the spatial distribution of the targets, there were two primary conditions of interest: those when the targets were in the same hemifield (SH) and those when the targets were in opposite hemifields (OH). These two conditions comprised 12 spatial arrangements, four for SH, and eight for OH. The four in the SH condition were each repeated, in order to match the number of trials in the opposite side condition. Each spatial arrangement was presented twice (four times for the SH condition), once probing the target at the end, and once probing the distractor at the end. The total was 32 trials—eight on each side of the screen in each spatial condition (targets on same or opposite side of screen). There were also 16 trials presented in the control “stationary” condition, eight in which the targets were on the same side of the screen, and eight where they were on opposite sides. For this control condition, the decision as to whether to probe the target or the distractor was randomly determined for each trial. One control subject was missing data from the stationary control condition, and was replaced with the NC group mean in the analysis using these data. When possible, the entire testing block was repeated so that double the trials were obtained, in order to enhance reliability (10 PD participants and 11 NC participants received double the trials in this way, and there was no difference in overall accuracy between those who did and did not complete double the trials).

### Eye tracking

Eye tracking procedures were the same as in Experiment 1.

## Experiment 2: Results

### PD vs. NC

**Fig 5:**
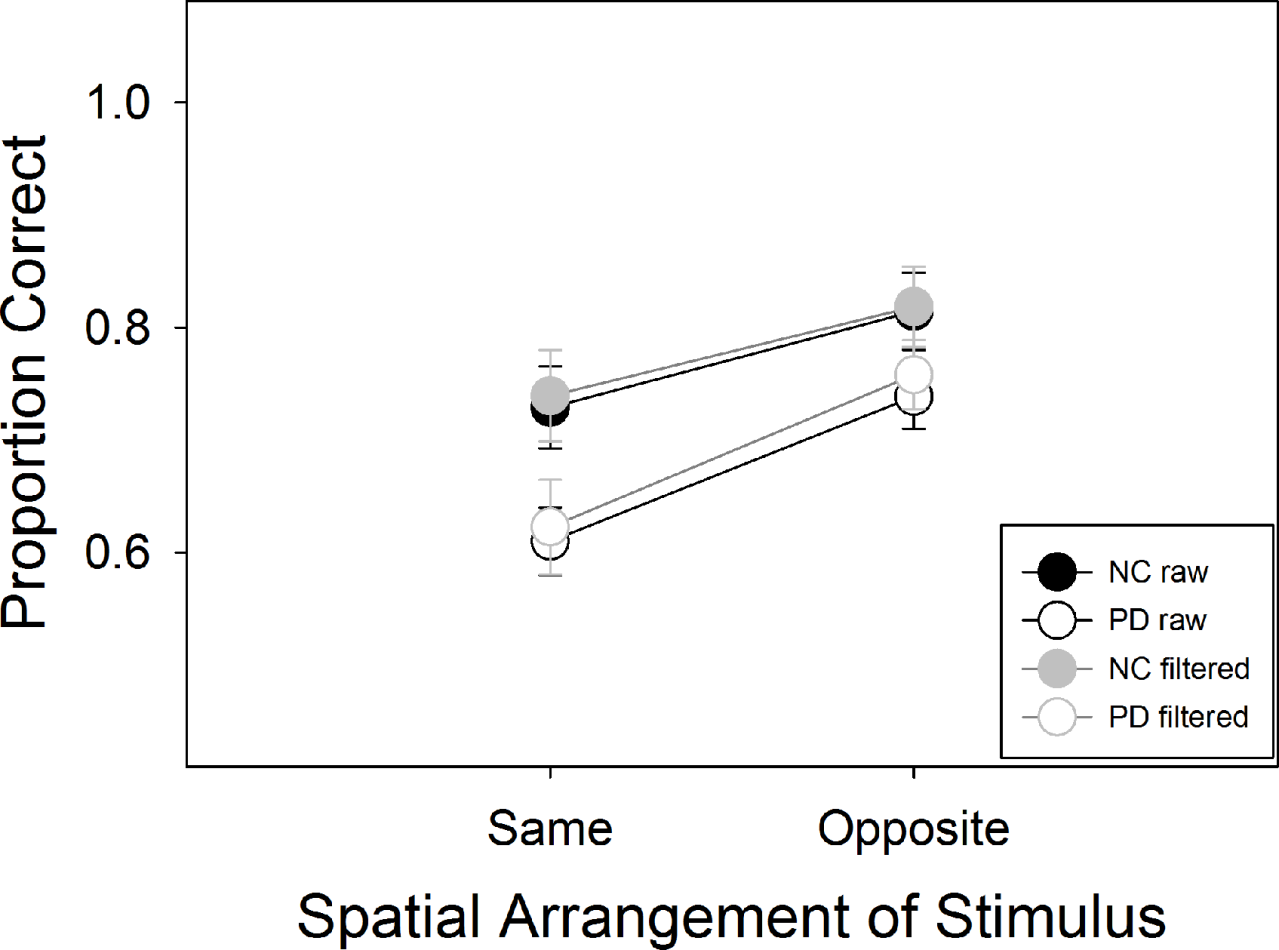
Results of Experiment 2. PD were less accurate than NC overall in both the raw dataset, and the filtered one based only on trials where subjects maintained fixation.

A two-way ANOVA with group and spatial condition as factors showed a main effect for group, F(1, 38) = 5.88, p = .02, η^2^ = .13, as well as spatial condition, F(1, 38) = 20.073, p < .001, η^2^ = .35, but no interaction between the two, F (1, 38) = .82, p = .37, η^2^ = .02 (Fig 5). To control for the effects of the stationary condition, we conducted separate one-way ANOVAs on the SH and OH spatial conditions, using the corresponding spatial condition in the stationary condition as a covariate. PDs exhibited worse performance than the control group in the SH condition (Group effect: F(1,36) = 4.45, p = .042, η^2^ = .11), but not for the OH condition (Group F(1,36) = .64, p = .43, η^2^ = .02). Analyzing the still condition in its own right, subjects performed better on the OH condition F(1,37) = 4.1, p = .05, η^2^ = .1, but there was no group difference F(1,37) = 2.31, p = .14, η^2^ = .06, and no interaction F(1,37) = .046, p = .83 η^2^ = .00.

**Fig 6:**
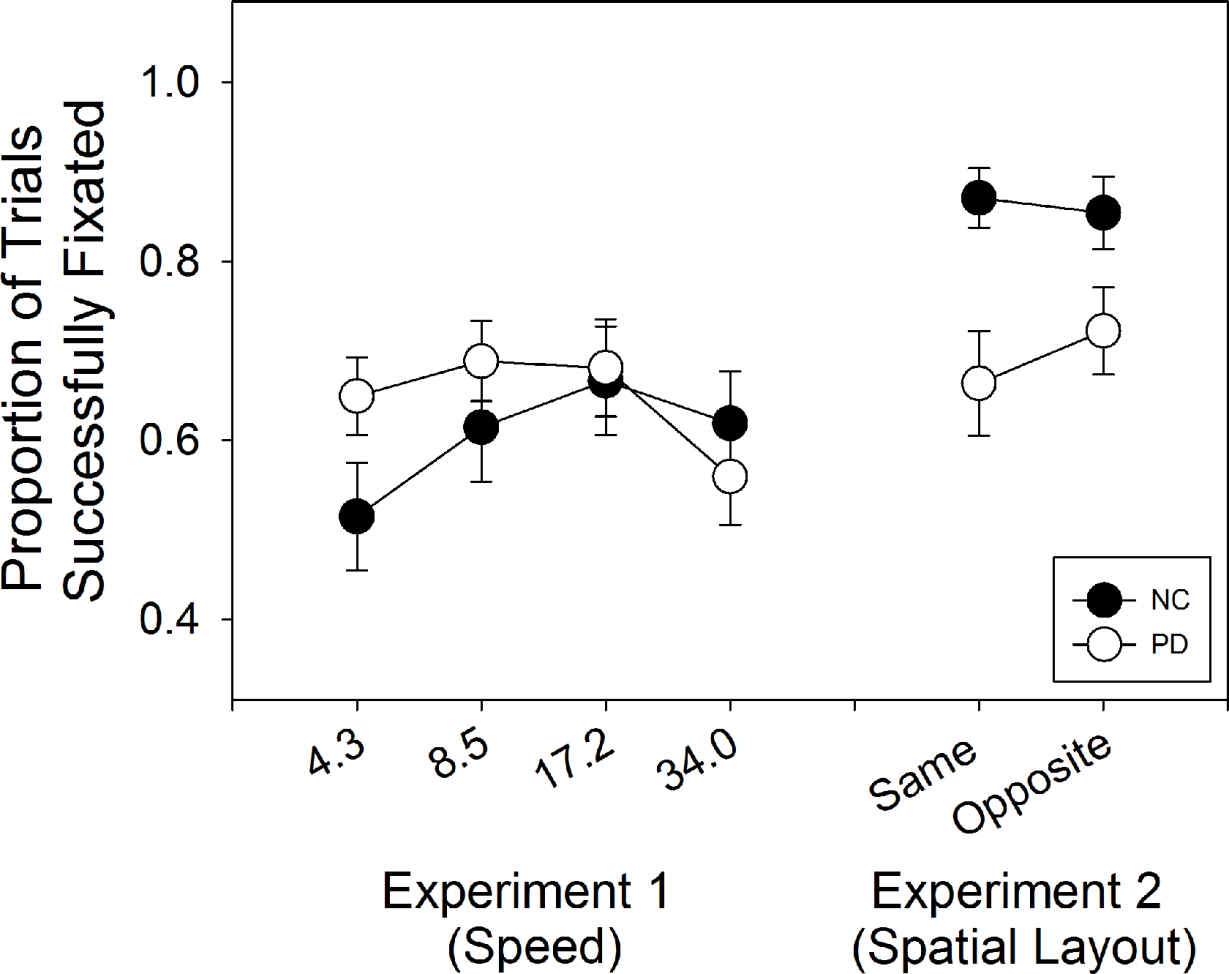
Proportion of trials fixated in Experiments 1 and 2. In Experiment 1, a significant interaction between group and stimulus speed emerged, and in Experiment 2, a main effect of group was present, with PD fixating successfully less frequently than NC.

Individuals with PD had less success maintaining fixation than controls (Fig 6). An ANOVA on group and spatial layout of the stimulus showed that PD maintained fixation less than NC, F(1,38) = 5.29, p = .027, η^2^ = .12. There was no effect of spatial layout, (F, 1, 38) = 1.79, p = .19, η^2^ = .05, but the interaction between spatial arrangement and group was significant, F(1,38) = 5.76, p = .02, η^2^ = .13. PD showed more successful fixations when the two targets were in opposite hemifields than when they were in the same hemifield, paired samples t-test, t(24) = 2.7, p = .013, and NC showed the opposite pattern, but not significantly so (t(14) = -0.95, p = .36).

We repeated the analysis on accuracy using only the trials for which participants did not break fixation. Data represented by fewer than 4 trials were considered missing, and replaced using multiple imputations as described in the methods and in Experiment 1. The number of data in these analyses that were missing and then replaced in this way was 2.5%. We ran 25 ANOVA’s on the 25 imputed datasets, and report the mean results of those analyses, which were similar to those of the unfiltered data. There was a significant effect of spatial condition, mean F = 10.0 (95% CI 9.52–10.47), mean p = .004 (95% CI .003–.004), mean η^2^ = .23 (95% CI .19–.27), and for group, mean F = 4.20 (95% CI 3.87–4.52), mean p = .044 (95% CI .041–.047), mean η^2^ = .10 (.10 –.11), but no interaction between the two, mean F = .73 (95% CI .64–.82), mean p = .41 (95% CI .38–.44) mean η^2^ = .019 (95% CI .016–.021).

### Correlations

Within the PD group, individuals’ accuracy in the SH and OH conditions was compared with their speed threshold from Experiment 1, to explore the extent to which performance in the two conditions reflected the same or different mechanisms. Speed thresholds did not correlate significantly with accuracy in the SH condition, r(25) = -.23, p = .28, but did correlate with accuracy from the OH condition, r = -.69, p < .001. These two correlations differed significantly according to Meng’s Z test (Z_22_ = 2.54, p = .011), suggesting that speed threshold related to performance on the OH, but not the SH condition. Comparing accuracy in the PD group in the the SH and OH conditions directly, there was no correlation for the raw, r(25) = .31, p = .13, or filtered, r(25) = -.08, p = .70, data. The results of this analysis changed when using the filtered dataset. When using only trials where fixation was maintained, SH accuracy from Experiment 2 was again not correlated with the threshold from Experiment 1, r(25) = -.17, p = .42, but this time neither was OH at r(25) = -.19, p = .36, and the two slopes did not differ from each other (Z = .06, p = .95). These results imply when subjects maintained fixation, performance in tracking multiple items in PD was similarly unrelated to speed threshold in Experiment 1, whether in the SH or OH condition.

Clinical variables were not related to accuracy data in Experiment 2. Accuracy in the SH condition was not correlated with total score from the UPDRS motor exam (r(22) = .20, p = .38), or Hoehn & Yahr stage (r(25) = .13, p = .54). Accuracy in the OH condition was also not correlated with the UPDRS motor exam total (r(22) = -.16, p = .48) or the Hoehn & Yahr stage (r(25) = -.04, p = .85). Furthermore, OH and SH were not correlated with BDI or BAI scores in the PD group (-.03 < r < .12, p > .60).

### LPD analysis

We examined LPD as a separate group and compared them to RPD and NC to see if attentional problems in the left hemifield particularly would emerge in LPD. LPD and RPD did not differ on any of the demographic variables shown in Table 1, or in clinical severity as measured by the UPDRS (p = 0.594). If there were indeed neglect-like attention problems in LPD, we would expect to find an interaction between group and side of the screen, with LPD being more impaired when targets were on the left. The interaction between side and group was not significant, however, F(2,37) = 1.0, p = .37, η^2^ = .05, nor was the interaction of side, spatial configuration and group, F(2,37) = .30, p = .74, η^2^ = .02.

We also examined the correlation between the extent to which PD participants performed in a neglect-like manner (accuracy difference in each condition subtracting trials where stimulus was on the right minus trials where the stimulus was on the left) and the extent to which their motor symptoms indicate left-severity/asymmetry. Of the 12 correlations (shown in Table 2), two had p values below .05, but were not significant when correcting for multiple comparisons.

**Table 2:** Relation between asymmetry of currnet motor symptoms and asymmetry of attentional performance. When correcting for 12 comparisons, none of the correlations below are significant, consistent with the analysis based on side of symptom onset reported in the text.

Condition	Asymmetry of current motor symptoms (positive values indicate predominance of left-symptoms.	Left-motor-symptom severity
Exp 1: Speed 1 R-L accuracy	r(23) = .05, p = .8	r(23) = .09, p = .68
Exp 1: Speed 2 R-L accuracy	r(23) = -.01, p = .97	r(23) = .05, p = .82
Exp 1: Speed 3 R-L accuracy	r(23) = -.13, p = .56	r(23) = -.04, p = .85
Exp 1: Speed 4 R-L accuracy	r(23) = -.47, p = .02	r(23) = -.46, p = .03
Exp 2: SH R-L accuracy	r(22) = -.37, p = .09	r(22) = -.34, p = .12
Exp 2: OH R-L accuracy	r(22) = .17, p = .45	r(22) = -.02, p = .95

## Discussion

The present study represents the first examination of sustained attention in PD to our knowledge. Results demonstrate that PD affects the ability to track a single target (Experiment 1) as well as the ability to track multiple targets (Experiment 2). PD and NC also showed differing patterns of oculomotor behavior during the task.

The fact that the deficit in PD in Experiment 2 remained significant when filtering out trials where fixation was not maintained suggests that a deficit of sustained attention exists in PD independently from oculomotor difficulties. On the other hand, in Experiment 1, there was no longer a group difference when using trials where subjects maintained fixation, suggesting that the deficit in PD was intimately tied to eye movements. It is tempting to infer that in Experiment 1, the PD performance deficit in this attention task should be attributed to faulty eye movements such as the inability to inhibit saccades toward the target. However, this would be rather speculative for two reasons. First, gazing nearer to the stimulus was not robustly related to accuracy in either group, either positively or negatively. Second, even if a strong correlation had existed, it is possible that the relationship is spurious, and a neural mechanism that is common to both the oculomotor system and attentional systems is compromised in some patients and manifests as altered oculomotor behavior as well as deficient performance outcomes.

There are several potential neural explanations for impaired performance by individuals with PD on the two experiments. First, the substantia nigra (SN), which is the primary target of cell death in PD leading to the characteristic motor symptoms of the disease, is directly connected to the superior colliculus (SC) [35,36]. The SC is important not only for determining eye movements but also for maintaining a map of salient stimuli in the environment [37,38]. SN pars reticulata (SNr) appears to project primarily to the intermediate and deep layers of the SC [35,36]. While it is these layers that are involved in generating saccades, some neurons in these layers appear to be responsible for simply selecting a visual stimulus, rather than preparing a motor movement toward it. The SNr, and basal ganglia in general, are thought to selectively inhibit SC activity, in order to filter some of the abundant information that is present in SC from its multiple inputs. The role of neurons in SC during the MOT task is unclear, but it seems likely that if SC activity is not properly inhibited/filtered with the aid of SNr input, attention would suffer, even independently of eye movements. Another possible neural substrate for the PD performance deficit shown in the present study relates to the fact that PD has been associated with reduced grey matter density in parietal cortex [14]. This area is also critical for spatial attention [23,39]. Particularly relevant to the current tasks, the posterior parietal cortex has been shown to be crucial for indexing multiple attentional targets (but is not sensitive to changes in speed of the targets). If neural networks in this area are disrupted, performance on attention tasks should be affected in a way similar to what the present results demonstrate. Finally, according to the Premotor Theory of Attention [40], moving the focus of attention is like a motor action (moving the eyes) and requires pre-motor planning. This idea is at least partly supported by fMRI studies demonstrating that premotor areas like FEF are recruited for spatial attention tasks [17]. It is possible that damage to FEF, or midbrain areas that connect to it affect both oculomotor response and attentional representation.

When controlling for accuracy on the still-condition in Experiment 2, individuals with PD showed a deficit only for the SH condition. Since resources are strongly independent for tracking objects in the left and right hemifield, the OH condition can be viewed as similar to the task in Experiment 1, except that each hemifield is engaging in the task at the same time. The SH condition is qualitatively different, however, since multiple objects are being tracked by one hemifield. Prior work has demonstrated that a broad fronto-parietal brain network is recruited when tracking one moving object, while only a subset of this network is sensitive to the number of objects tracked [16]; that is, the brain networks supporting MOT differ from those supporting single object tracking (SOT) [17]. In the present study, there was a strong correlation among participants with PD between performance in the SOT of Experiment 1 and performance in tracking one object per visual hemifield in Experiment 2 (Condition OH); however, there was no correlation between the results of Experiment 1 and performance in tracking two objects within a single visual hemifield (condition SH). The slopes of these two correlations differed significantly in PD, suggesting that the critical resource bottleneck differs between the SH and OH conditions. We suggest that in the SH condition, the priority map [41] that represents the spatial location of items of interest is the critical bottleneck, whereas in the OH condition and SOT condition, a more central attentional structure exhibits modest resource limitation. It is also possible that one or the other of the two performance deficits in PD is more closely linked to SC dysfunction, while the other is more aligned with parietal dysfunction. Moreover, our results suggest that in PD both the priority maps and the central attentional mechanisms are impaired. Both of these mechanisms may be important in causing real world problems for patients in situations where visuospatial attention demands are high, such as driving.

The results shed light on the understanding of hemineglect in PD [42]. Deficiencies in applying attention to the left side of space would seem to be one possible mechanism underlying hemineglect-like performance which has been shown in patients with LPD. The lack of a differential impairment in the left hemifield (or overall, for that matter) in LPD in this study suggests that attention of the sort indexed by the present task is unlikely to underlie any neglect syndrome that may occur in LPD.
